# First report of the cactus cyst nematode, *Cactodera cacti,* from a cactus garden in Idaho

**DOI:** 10.21307/jofnem-2019-044

**Published:** 2019-07-23

**Authors:** Andrea M. Skantar, Zafar A. Handoo, Mihail R. Kantor, Maria N. Hult, Saad. A. Hafez

**Affiliations:** 1 Mycology and Nematology Genetic Diversity and Biology Laboratory, USDA, ARS, Northeast Area, Beltsville, MD, 20705; 2 University of Idaho, Parma, ID, 83660

**Keywords:** *Cactodera cacti*, Cactus, Idaho, Nematodes

## Abstract

In April 2018, a cyst nematode was discovered from soil samples collected from a cactus garden collection in Meridian, Ada County, Idaho, USA. The cactus garden collection field reported was observed with localized areas of heavily stunted plants. Roots from affected plants displayed moderate numbers of nematode cysts. Living nematode juveniles (J2) recovered from the cysts were examined morphologically and molecularly for species identification which indicated that the specimens were *Cactodera cacti*. This is the first report of the cactus cyst nematode, *C. cacti* in Idaho.

The cactus cyst nematode, *Cactodera cacti* (Filipjev and Schuurmans Stekhoven, 1941; Krall amily Heteroderidae on the basis of th and Krall, 1978) is distributed worldwide, mainly on plants of family Cactaceae grown in glasshouses as ornamentals. This cyst nematode has been well known for over 85 years and was first recorded and described by Adam (1932) from Maartensdiijk, near Utrecht, The Netherlands. The dispersal of *C. cacti* from native regions in the America is believed to be associated with the international trade of infested ornamental cactus plants around the world (Subbotin et al., 2010). In April 2018, a cyst nematode was discovered from soil samples collected from a cactus garden collection in Meridian, Ada county, Idaho, USA. The sample was sent to one of us (SH) who in turn forwarded the extracted cysts to the Mycology and Nematology Genetic Diversity and Biology Laboratory (MNGDBL) for identification purposes. The cactus garden collection field reported was observed with localized areas of heavily stunted plants. Roots from affected plants displayed a moderate number of nematode cysts.

## Material and methods

### Various stages

Cysts, white females, second-stage juveniles (J2), and eggs were obtained from soil and roots associated with cactus plants from the cactus collection in Meridian, Idaho, USA. Juveniles for morphological observations were separated from soil by sieving and using Baermann funnel extraction, or were recovered from cysts removed from fresh roots and kept in water in watch glasses. Juveniles were fixed in 3% formaldehyde and processed to glycerin by the formalin glycerin method (Golden, 1990; Hooper, 1970). Females and some cysts were typically removed from roots after fixation for 12 hr in 3% formaldehyde solution. Photomicrographs of cyst vulval cones, females, and J2 were made with an automatic 35-mm camera attached to a compound microscope having an interference contrast system. Roots and whole cysts were photographed under a dissecting microscope, and light microscopic images of fixed nematodes were taken on a Leica WILD MPS48 Leitz DMRB compound microscope. Measurements were made with an ocular micrometer on a Leica WILD MPS48 Leitz DMRB compound microscope. All measurements are in micrometers, unless otherwise stated.

**Figure 1:**
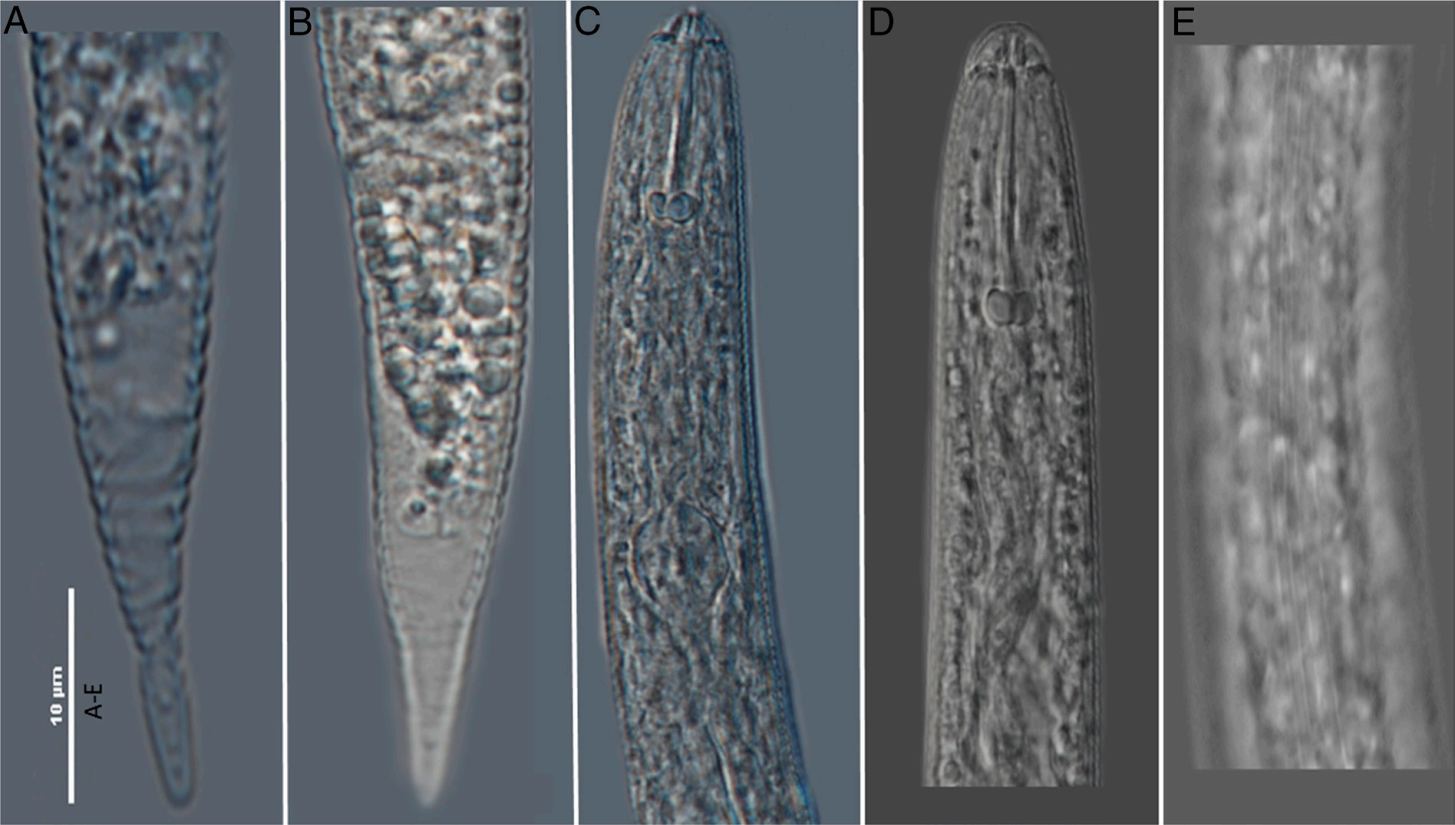
Photomicrographs of second-stage juveniles (J2) of *Cactodera cacti*. (A-B) tails; (C-D) head; (E) lateral field.

**Figure 2:**
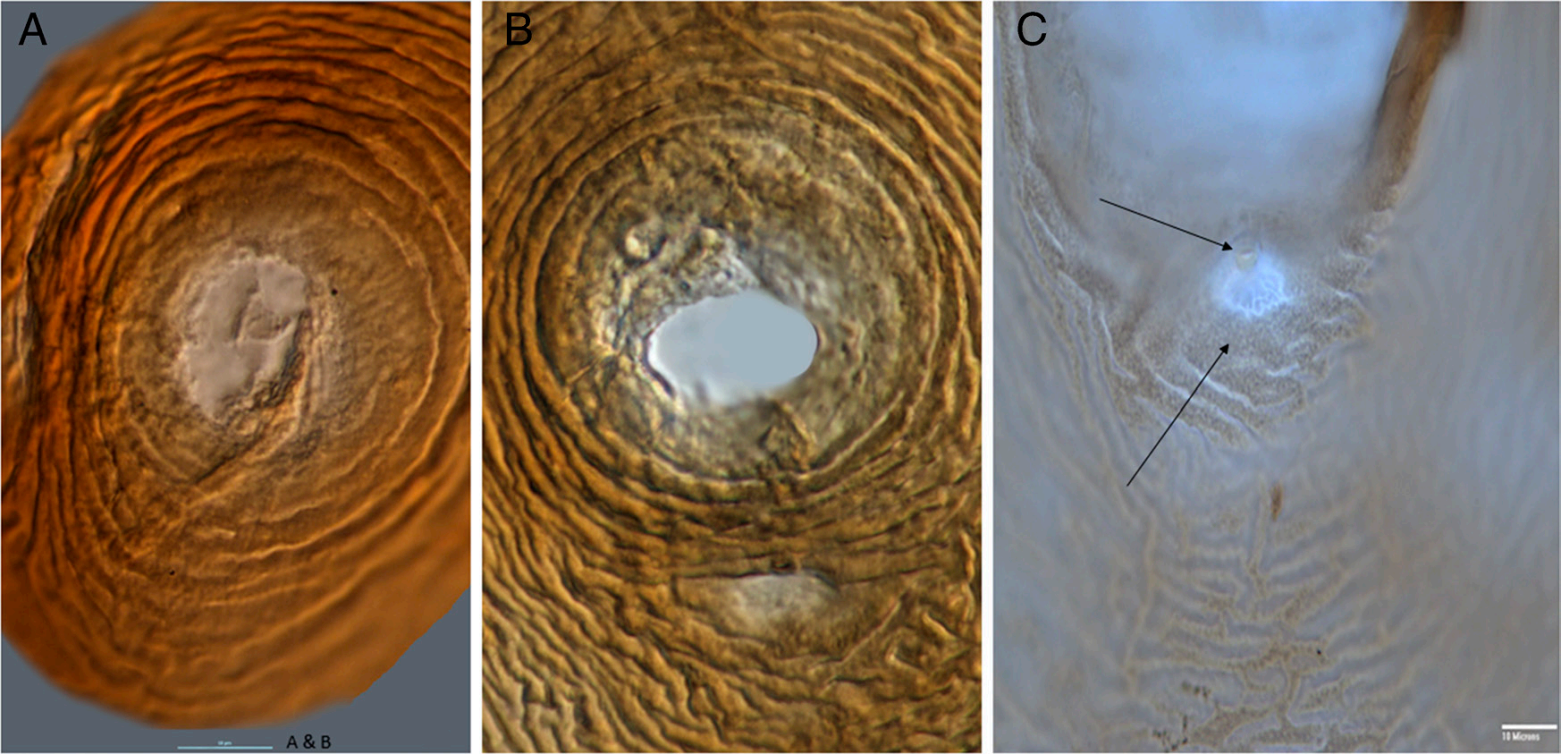
(A–C) Photomicrographs of vulva cones of *Cactodera cacti*. A-B scale bar = 50 µm and C = 10 μm; C arrows showing the anal area and punctations.

Living nematode juveniles (J2) recovered from the cysts were examined morphologically and molecularly for species identification at the MNGDBL. Observations of morphological characters critical for identification (Fig. 1A–E and Fig. 2A–C) indicated that the specimens were *Cactodera cacti* (1–3).

The ITS 1&2 rDNA region was amplified with primers TW81 and AB28 (Joyce et al., 1994) and conditions as described previously (Skantar et al., 2012), producing PCR amplicons of 985 bp. The PCR products were cleaned with the Monarch DNA Gel Extraction Kit (NEB, Ipswitch, MA) and then cloned using the Strataclone PCR Cloning Kit (Agilent, Santa Clara, CA). Six ITS rDNA clones representing three J2 were prepared with the Monarch Plasmid Miniprep Kit (NEB) and sequenced by Genewiz, Inc. (MH477533-MH477538). The 28 S rDNA D2-D3 expansion segment was amplified using primers D2A and D3B (DeLey et al., 1999) and conditions as described previously (Skantar et al., 2012). Hsp90 sequences were amplified with primers U288 and L1110 and gave products of 1219 bp. These were cloned and sequenced as described above and submitted to GenBank under accession numbers MH484605-MH484607.

**Figure 3:**
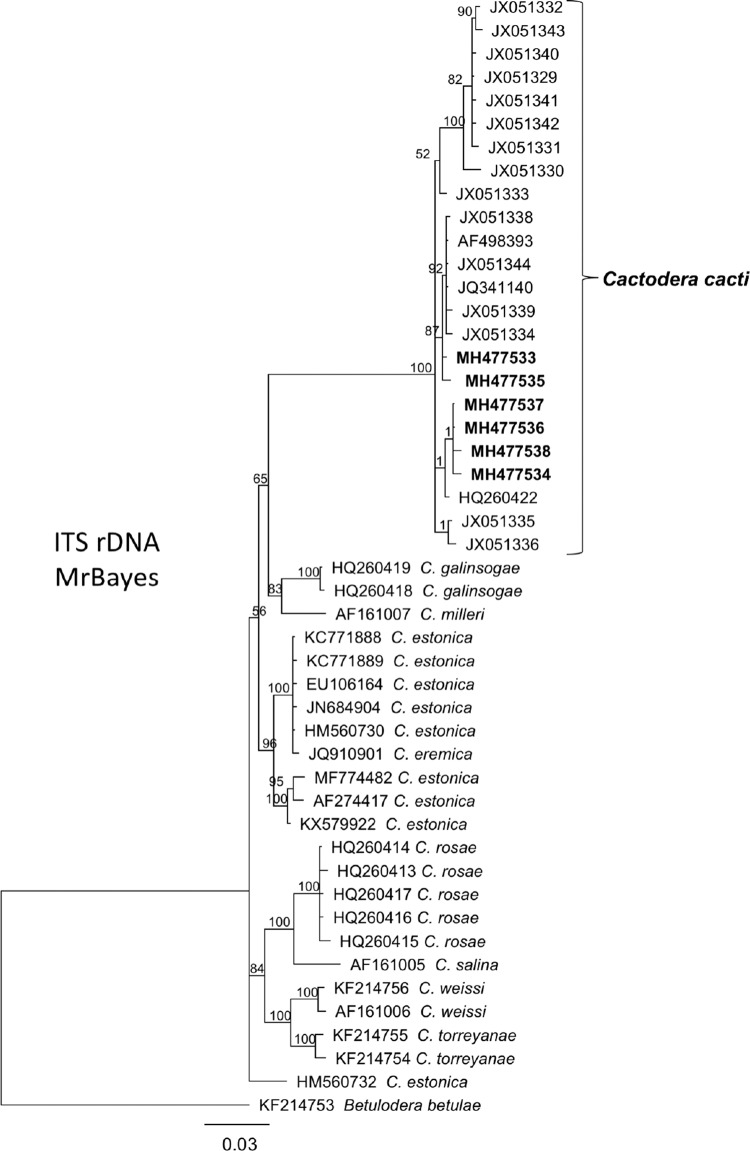
Phylogenetic relationships of *Cactodera cacti* among other species and populations within *Cactodera,* as inferred from an alignment of ITS rDNA. A 50% majority rule consensus tree from Bayesian analysis was generated from two runs as inferred from alignment of ITS rDNA using the GTR + I + G model of nucleotide substitution. Branch support values above 50% are shown. New sequences are highlighted in bold type on appropriate branches.

Separate alignments of ITS rDNA and Hsp90 genomic DNA sequences were constructed using the MAFFT algorithm within Geneious v. 10.2.6. For ITS, the best-fitting model of nucleotide substitution GTR + I + G was estimated using jModelTest based on the Akaike Information Criterion. Phylogenetic relationships were estimated with Bayesian interference (BI) on the CIPRES Science Gateway (www.phylo.org/; Miller et al., 2010). The parameters for BI analyses of ITS rDNA were implemented in CIPRES as described in Skantar et al. (2012), with a random starting tree, two independent runs with four chains (1.0 × 10^6^ generations). Markov chains were sampled at intervals of 500 generations and burn-in of 10,000). The 50% majority rule consensus tree (Fig. 3) was generated with posterior probabilities (PP) calculated for each clade. The Hsp90 genomic alignments were used to infer phylogenetic relationships in CIPRES under the model GTR + I + G as determined in jModelTest and implemented in MrBayes as described above. Separate trees based upon alignments of exon regions only were also constructed with separate partitions for 1st, 2nd, and 3rd codon positions (not shown).

## Description

### Measurements

Measurements of second-stage juveniles (*n* = 10) included length of body (range = 415–478 μm, mean = 449.3 μm), stylet well developed (22.5–25.0 μm, 23.3 μm) with semi-rounded basal knobs, convex anteriorly and posteriorly, tail (35.0–50.0 μm, 42.0 μm), and hyaline tail terminus (14–25 μm, 18.3 μm). The lateral field had four distinct lines. Shapes of the tail, tail terminus, and stylet knobs were also consistent with *C. cacti* except for some variations noted in this population showing some tail with peg-like terminus with a constriction in hyaline area of tail terminus. The cysts (*n* = 10) were basically lemon shaped, abulate, circumfenestrate, light to dark or reddish brown in color and had a straight to wavy line type of cyst wall cuticular pattern (Fig. 2A–C); anus opening was prominent; punctations often present in terminal area of cyst; morphometrics of cysts were also consistent with *C. cacti*.

### Molecular analysis

The ITS rDNA sequences from this population varied from 0 to 7 bp among each other. Intraspecific variation among all available ITS sequences of *C. cacti* ranged from 0 to 2.6% and interspecific variation with *C. estonica* ranged from 9.9 to 11.7%. The ITS rDNA sequences from the Idaho population formed a strongly supported clade with 18 other sequences of *Cactodera cacti* (Fig. 3).

Three 28 S rDNA amplicons of 721 bp were obtained from three separate J2 and gave rise to identical sequences (MH478572-MH478574). MegablastN search of the NCBI NR database showed 99% identity to a single sequence of *C. cacti* (DQ328702) from Germany but also to several *C. estonica* sequences (KX230467-KX230473), indicating that 28 S is unable to clearly resolve the identity of the population.

**Figure 4:**
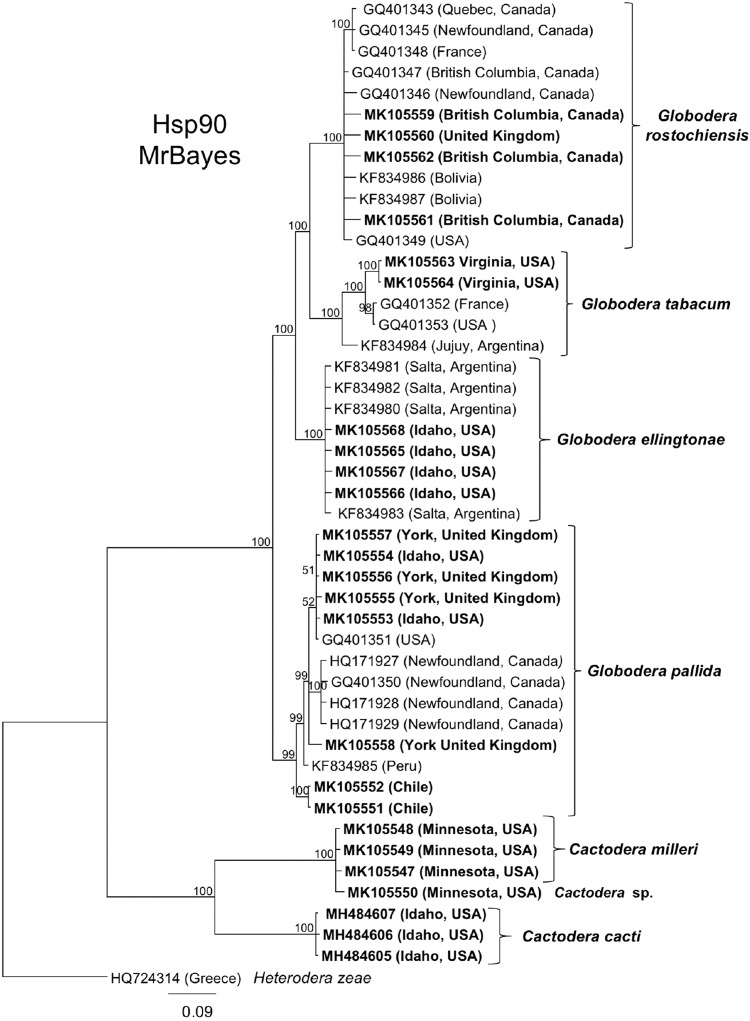
Phylogenetic relationships of *Cactodera cacti* among other species and populations of cyst nematodes, as inferred from an alignment of partial Hsp90 genomic DNA. A 50% majority rule consensus tree obtained from Bayesian analysis was generated using the GTR + I + G model of nucleotide substitution. Branch support values above 50% are shown on appropriate branches. New sequences are highlighted in bold type.

Partial Hsp90 sequences were aligned with selected sequences from other *Cactodera* spp. and other cyst nematodes as available. Hsp90 sequences were not available from other *C. cacti* isolates, so the closest DNA match was to sequences from *Cactodera milleri*, *Heterodera zeae*, and to a lesser degree to several *Globodera* spp. sequences. Hsp90 from the Idaho population formed a strongly supported clade that grouped separately from *C. milleri* and an unnamed population of *Cactodera* sp. (Fig. 4). While not the focus of this identification, it is notable that the newly acquired Hsp90 genomic sequences from *Globodera pallida*, *G. rostochiensis*, *G. tabacum*, and *G. ellingtonae* formed distinct clades that were consistent with those determined from Hsp90 by Lax et al. (2014). The acquisition of additional Hsp90 sequences from cyst nematode species and populations should help to further strengthen cyst nematode relationships inferred from ITS rDNA.

Based upon this collective morphological and molecular data, we identify this isolate as *Cactodera cacti.* To our knowledge this is the first report of the cactus cyst nematode, *Cactodera cacti* in Idaho.
